# One size fits null: attentional brain responses differ depending on insomnia subtype

**DOI:** 10.1093/sleep/zsaf056

**Published:** 2025-05-22

**Authors:** Wenrui Zhao, Eus J W Van Someren, Glenn J M van der Lande, Sjors van de Ven, Frank J van Schalkwijk, Tessa F Blanken, Jennifer R Ramautar, Roy Cox

**Affiliations:** Sleep Medicine Center, Chongqing Traditional Chinese Medicine Hospital, Chongqing, 400021, China; Department of Sleep and Cognition, Netherlands Institute for Neuroscience, An Institute of the Royal Netherlands Academy of Arts and Sciences, Amsterdam, The Netherlands; Department of Sleep and Cognition, Netherlands Institute for Neuroscience, An Institute of the Royal Netherlands Academy of Arts and Sciences, Amsterdam, The Netherlands; Department of Psychiatry, Amsterdam Public Health Research Institute and Amsterdam Neuroscience Research Institute, Amsterdam UMC, Vrije Universiteit, The Netherlands; Department of Integrative Neurophysiology, Center for Neurogenomics and Cognitive Research (CNCR), Amsterdam Neuroscience, Vrije Universiteit Amsterdam, The Netherlands; Coma Science Group, GIGA-Consciousness, University of Liège, Liège, Belgium; Centre du Cerveau2, University Hospital of Liège, Liège, Belgium; Department of Sleep and Cognition, Netherlands Institute for Neuroscience, An Institute of the Royal Netherlands Academy of Arts and Sciences, Amsterdam, The Netherlands; Department of Biological Psychology, Vrije Universiteit Amsterdam, The Netherlands; Department of Sleep and Cognition, Netherlands Institute for Neuroscience, An Institute of the Royal Netherlands Academy of Arts and Sciences, Amsterdam, The Netherlands; Hertie-Institute for Clinical Brain Research, Center for Neurology, University Medical Center Tübingen, Otfried-Müller Str. 27, 72076 Tübingen, Germany; Department of Psychology, University of Amsterdam, The Netherlands; Department of Sleep and Cognition, Netherlands Institute for Neuroscience, An Institute of the Royal Netherlands Academy of Arts and Sciences, Amsterdam, The Netherlands; N=You Neurodevelopmental Precision Center, Amsterdam Neuroscience, Amsterdam Reproduction and Development, Amsterdam UMC, Amsterdam, The Netherlands; Child and Adolescent Psychiatry and Psychosocial Care, Emma Children’s Hospital, Amsterdam UMC, Vrije Universiteit Amsterdam, Amsterdam, The Netherlands; Emma Center for Personalized Medicine, Amsterdam UMC, Amsterdam, The Netherlands; Department of Sleep and Cognition, Netherlands Institute for Neuroscience, An Institute of the Royal Netherlands Academy of Arts and Sciences, Amsterdam, The Netherlands

**Keywords:** insomnia disorder, insomnia subtypes, auditory oddball, event-related potentials

## Abstract

**Study objectives:**

Event-related potential (ERP) studies on attentional brain processes in insomnia disorder (ID) have yielded inconsistent findings. Such inconsistencies may relate to small sample sizes, limited corrections for multiple comparisons, and the possibility of heterogeneity within the clinical population. We aimed to overcome these limitations by studying ERP responses both across and within subtypes in a larger sample of ID.

**Methods:**

ERPs were recorded in 201 participants with ID and 70 normal sleeper controls (NS) with an auditory oddball task. Participants with ID were subtyped using a validated multivariate trait profile. Analyses evaluated subtype-specific and nonspecific deviations using both conventional ERP components as well as cluster-based permutation tests.

**Results:**

All five subtypes were well-represented in the ID sample (subtypes 1–5 respectively *N* = 31, 83, 28, 29 and 19). ERP component analyses with false discovery rate corrections revealed no evidence for differences between the heterogeneous ID group and NS. However, subtype-specific analyses revealed that ERPs were significantly altered, but in different ways for different subtypes. Specifically, ERP component analyses revealed stronger N100 amplitudes for standards and deviants both in subtypes 2 and 3, and a lower P300 amplitude and longer P300 latency for deviants in subtype 3. Cluster-based permutation tests on ERPs corroborated the P300 amplitude effect for deviants in subtype 3, with subtype 3 and 4 additionally showing a smaller difference between deviant and standard P300 amplitudes.

**Conclusion:**

Our findings indicate that ID is a heterogeneous disorder. Ignoring subtype identity dilutes ERP alterations occurring only in specific insomnia subtypes.

## Graphical Abstract

**Graphical Abstract:**
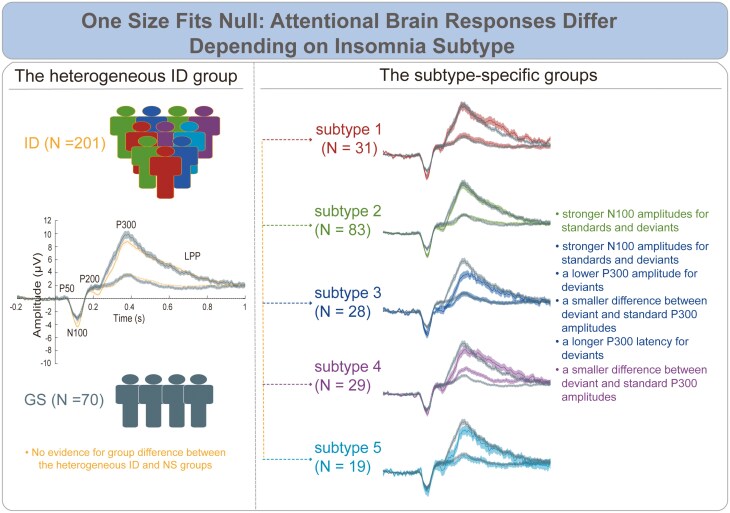

**Statement of significance**

Given the challenges posed by the heterogeneity within insomnia disorder, inconsistencies have been observed across ERP studies on attentional brain processing. Thus, we conducted a comprehensive assessment of ERP responses both across and within insomnia subtypes in a larger sample of participants. Our findings yielded no ERP evidence for differences between heterogeneous insomnia and normal sleepers. However, ERP deviations appear more clearly when considering different subtypes in isolation. Moreover, individual subtypes show additional deviations that were diluted to no significance in the heterogeneous group. These findings indicate that ERPs are altered in different ways for different insomnia subtypes. Pooling across heterogeneous insomnia patients without differentiating subtypes dilutes deviations presenting only in specific subtypes. Subtyping seems required to better understand brain mechanisms of insomnia vulnerability.

Insomnia disorder (ID) is characterized by self-reported poor sleep quality, including difficulties initiating and/or maintaining sleep, and/or waking up earlier than desired [1]. Estimates of ID prevalence in the general population range from 12% to 20% [2]. People with ID subjectively experience difficulties with daytime mood, physical well-being, and cognitive functioning, e.g. attention, alertness, and memory [3]. A meta-analysis suggests moderately affected objective performance in some cognitive domains including attention [4]. Nevertheless, the neural correlates underlying the observed cognitive dysfunction remain unclear and require further investigation.

Most theoretical models on insomnia assign key importance to maladaptive arousal affecting cognitive, cortical, and/or physiological domains [5–8]. Increased electroencephalographic (EEG) beta power has been observed “round-the-clock” across sleep and wakefulness and is recognized as physiological evidence supporting hyperarousal theories [5, 9, 10]. While the neurocognitive model of insomnia [7] proposes that hyperarousal may correspond to increased sensory processing and/or reduced inhibitory processing, findings of EEG studies on hyperexcitability and/or inhibitory deficits in ID are far from consistent.

One approach to assess attentional processing is the auditory oddball paradigm. In this paradigm, EEG is recorded while frequent standard tones are occasionally interrupted by deviant (or oddball/rare) tones. The average EEG responses to these tones, commonly called event-related potentials (ERPs), have been suggested to be altered in ID [11]. However, as shown in Table 1, studies investigating oddball ERPs have yielded quite inconsistent findings. Inconsistencies across these studies might relate to the wide variability in the specific ERP metrics reported to be affected, including amplitudes and latencies for different ERP components (e.g. N100, P200, P300); different task settings (e.g. recording before and after sleep); different stimulus conditions (standard, deviant); and EEG channels analyzed (ranging from 1 to 11 channels). In addition, most studies employed small samples with limited correction for multiple comparisons, increasing the probability of false positives. Consequently, it remains unclear which aspects of the oddball ERPs are consistently altered in ID, if any.

**Table 1.** Summary findings of event-related potentials during wakefulness in insomnia disorder based on auditory oddball task

Study [Author (y)]	N (gender) (ID/NS)	Age	Task settings	Electrodes	Measures	ERP components	Statistical methods	Findings (ID vs. Control)
Hull, 1993 [12] (dissertation)	9(6F)/8(6F)	ID: 20-31 NS: 19-45	Evening recording	Fz, Cz, Pz	Amplitude; latency	N100 (70-130ms); N200 (200-400ms); P300 (300-600ms)	mixed ANOVAs without corrections	No significant between-group difference
Anderer et al., 2003 [13]	49 F/48 F	MI: 46-67 NS: 46-68	Morning recording	Fz, Cz, Pz	Amplitude; latency	Standard: N100, P200; Deviant: N200, P300	Independent sample *t*-tests	Standard: P200 amplitude at Fz/Cz/Pz ↓ Deviant: P300 amplitude at Fz/Cz/Pz ↓; P300 latency↑
Devoto et al., 2003 [14]	11(7F)/11(5F)	ID: 22 ± 4 NS: 21 ± 1	Morning recordings after good and bad night	Fz, Cz, Pz	amplitude	P300 (200-500ms)	mixed ANOVAs without corrections	P300 amplitude ↑ (after bad night); P300 amplitude ↓ (after good night)
Devoto et al., 2005 [15]	7(4F)/7(4F)	ID: 21.0 ± 2 NS: 22.6 ± 2	Morning and evening recordings for one-week	Fz, Cz, Pz	Amplitude	P300 (200-500ms)	mixed ANOVAs without corrections	P300 amplitudes ↑ (only after the worst night both in the morning and evening)
Sforza and Haba-Rubio, 2006 [16]	15(10F)/13(7F)	Psy-I: 43.5 ± 9.4 NS: 40.2 ± 6.4	Morning and evening recordings	Fz, Cz, Pz	Amplitude; latency	N100 (70-140ms) P200 (120-220ms) P300 (250-500ms)	mixed ANOVAs without corrections	No significant between-group difference both in the morning and evening
Bastien et al., 2008 [17]	15(5F)/16(10F)	ID: 46.3 ± 7.6 NS: 36.8 ± 10.2	Morning and evening recordings for two nights	Fz, Cz, Pz	Amplitude; latency	N100 (60-110ms) P200 (120-200ms)	mixed ANOVAs with Bonferroni corrections	Standard: N100 amplitude at Cz (morning)/Pz (evening) on the second night ↑; N100 latency ↑; P200 latency ↑; Deviant: P200 latency ↓
Turcotte et al., 2011 [18]	Psy-I: 26(12F) Para-I: 26(20F) NS: 26(16F)	Psy-I: 42.7 ± 8.5 Para-I: 40.4 ± 9.0 NS: 37.0 ± 9.0	Evening recording	Cz	Amplitude; latency	N100 (70-150ms) P200 (150-250ms)	mixed ANOVAs with Bonferroni corrections; one-tailed *t*-tests	*t*-tests results in standard condition: P200 amplitude (Para-I) ↑; P200 latency (Psy-I) ↑
Kertesz and Cote, 2011 [19]	SOI: 13(10F) NS: 12 (9F)	SOI: 22.5 ± 7.2 NS: 22.6 ± 5.74	Morning and evening recordings for two nights	Frontal (F3, Fz, F4) Central (C3, Cz, C4) Parietal (P3, Pz, P4) Occipital (O1, O2)	Amplitude	N100 (75-150ms) P200 (150-250ms) P300 (250-450ms)	mixed ANOVAs without corrections	Deviant: P300 latency at Fz on the first night 1 ↑
Cortoos et al., 2014 [20]	13(NR)/12 (NR)	ID: 40.76 ± 9.4 NS: 45.36 ± 7.28	Evening recording	Fz, Cz	Amplitude; latency	N100 (70-140ms) P200 (160-280ms)	mixed ANOVAs without corrections	Deviant & Standard: P200 amplitude at Fz and Cz ↓
Aseem and Hussain, 2019 [21]	15(7F)/15 (6F)	ID: 19.9 ± 2.7 NS: 20.2 ± 3.9	Evening recordings after good and bad night	Cz	Amplitude; latency	P300	mixed ANOVAs without corrections	P300 latency for both good and bad night↑
Ding et al., 2023 [22]	33(17F)/34()	ID: 23.12 ± 2.74 NS: 23.27 ± 2.64	NR	Fz, FCz, Cz, Pz	Amplitude	N200 (220-280ms) P300 (350-450 ms)	mixed ANOVAs without corrections	P300 amplitude↓

Abbreviations: ID, insomnia disorder; NS, normal sleepers; MI, menopausal insomnia; NR, not reported; Psy-I, psychophysiological insomnia; Para-I, paradoxical insomnia; SOI, sleep onset insomnia.

A previously unaddressed yet critical factor to consider in the variability of results is that ID is likely not a single uniform disorder [23], but rather consists of different subtypes with distinct profiles of personality traits, life history, affect, and so on [24], Data-driven methods identified five stable ID subtypes of which the profiles can be summarized as (1) highly distressed, (2) moderately distressed but reward sensitive, (3) moderately distressed and reward insensitive, (4) slightly distressed with high reactivity, and (5) slightly distressed with low reactivity [24], More recently, these subtypes were shown to exhibit distinct profiles of structural brain connectivity [25]. Considering this, ERPs could be altered in different ways for different patterns of these features, and pooling across subtypes may dilute effects present in only specific insomnia subtypes. Depending on the recruitment strategy (e.g. from a psychiatric clinic vs. general population), previous studies likely differed in the proportions of subtypes in their heterogeneous samples of participants. This becomes especially problematic for smaller sample sizes, where it becomes increasingly likely that certain subtypes are overrepresented, underrepresented, or entirely absent. Hence, the aforementioned lack of replicability of ERP findings might be explained by the combination of small ID samples (Table 1: most *N* < 20) and heterogeneity. Indeed, Blanken *et al*. (2019) already provided a proof-of-principle by showing higher P300 and late positive potential (LPP) amplitudes to standard tones in subtype 4 but not in subtype 2 [24]. However, that study did not have sufficient EEG data to evaluate ERP responses across all five subtypes, leaving unanswered whether the remaining subtypes are also characterized by specific ERP patterns.

The current study investigated the hypothesis that ERP deviations may be specific to certain ID subtypes and the effects may be diluted when pooling subtypes into a heterogeneous sample. Building on the limited ERP findings reported in Blanken et al. (2019), we acquired additional EEG data to establish a large sample of ID and control participants that allowed for detailed subtype-specific analyses of ERP alterations. To achieve a comprehensive assessment of the ERP features and to control for multiple comparisons, we analyzed ERPs considering both conventional ERP components (P50, N100, P200, P300, LPP) and cluster-based permutation approaches. We also performed cluster-based time-frequency analyses (also known as event-related spectral perturbation, ERSP), as time-frequency analyses may uncover neural responses not captured by ERPs. Given that this is the first study investigating oddball EEG features potentially distinguishing ID subtypes, we did not have specific secondary hypotheses regarding how each neural feature might be affected in each subtype and particular findings should be considered exploratory.

## Methods

### Participants

Across different studies in our lab, ERPs were successfully acquired during an oddball task with an accuracy rate of ≥80% in 271 participants. Of them, 201 had a confirmed ID diagnosis according to the Diagnostic and Statistical Manual of Mental Disorders (DSM) and International Classification of Sleep Disorders (ICSD) criteria, while 70 qualified as normal sleepers (NS); All participants filled out the Insomnia Severity Index (ISI) [26]. Participants with current chronic use of medications, including hypnotics and psychotropics, were excluded. Detailed information about data inclusion and exclusion can be found in Figure S1. Individual study protocols were approved by the ethics committees of either VU Medical Center or University of Amsterdam. Participants gave written informed consent in accordance with the Declaration of Helsinki and were paid for participation.

### Task protocol

The auditory oddball task was presented using Eprime v2.0.10.242 (Psychology Software Tools, Pittsburgh, USA) during pre-sleep wakefulness in the evening. The task included two runs, where each run consisted of a sequence of 200 trials. Every trial started with a fixation cross presented for 1000 ms, followed by an auditory stimulus (standard or deviant tone) for 400 ms. Of the trials, 85% were lower pitch standard tones (170 trials, 1000 Hz), and 15% were higher pitch deviant tones (30 trials, 1200 Hz). The participants were asked to respond to both stimulus types by pressing response buttons using the index and middle fingers of the dominant hand. Mapping of stimulus type to response finger was counterbalanced across subjects. The time limit to respond was 1000 ms. The inter-stimulus interval was kept between 1200 and 1600 ms. Prior to the experimental runs, a training session of 20 trials was presented, and only after successful execution experimental runs proceed. For oddball performance analyses, we considered accuracy rates and reaction times both across stimulus types, and for the standard and deviant conditions separately.

### EEG assessment and preprocessing

High-density EEG was recorded using a 256 Ag/AgCl channel LTM HydroCel EEG net (Electrical Geodesic Inc, Eugene, OR) connected to a Net Amps 300 amplifier (input impedance: 200 MΩ, A/D converter: 24 bits, sample frequency 1000 Hz). The ground electrode was placed between CPz and Pz, with Cz serving as the online reference. Electrode impedances were kept below 100 kΩ.

EEG preprocessing was performed using EEGLAB 2021.0 and custom scripts running under Matlab 2020a [27]. Cheek and neck electrodes were removed reducing the number of channels to 184. Signals were then re-referenced to the averaged mastoids and bandpass filtered between 0.5-45 Hz. Independent component analysis (ICA) was used to detect artifacts such as eye blinks and eye movements and the EEGLAB plugin ICLabel was used to automatically label and reject components having ≥90% probability of being eye artifacts [28]. To enable manual signal quality review and make analyses more tractable, we included 11 channels previously used in the oddball literature (F3, Fz, F4, C3, Cz, C4, P3, Pz, P4, O1, O2) for subsequent ERP and time-frequency analysis. Noisy channels were detected by visual inspection of preliminary ERP plots and replaced by a neighboring sensor (30 of 271 participants had at least one noisy channel replaced).

To reduce boundary effects occurring at the start and the end of the trials, we initially extracted a larger time interval between −2000 and 4000 ms than the time period of interest used in ERP and time frequency analysis. Epochs exceeding a 150 μV threshold were omitted from the analyses, yielding an average of included epochs of 389.42 ± 13.64 (standard: 330.79 ± 11.67; deviant: 58.64 ± 2.34) for the ID group, and of 383.93 ± 41.75 (standard: 325.99 ± 35.53; deviant: 57.94 ± 6.27) for the NS group. These numbers did not differ significantly between groups (all *p* ≥ .10).

### ERP analysis

To fully evaluate the effect of ID and its subtypes on oddball ERP features, we performed both conventional ERP component analyses and cluster-based permutation approaches. Epochs were re-extracted from −200 to 1000 ms surrounding stimulus onset, and baseline-corrected from −200 to 0 ms. ERP components were separately defined for deviants and standards in accordance with previous studies. Specifically, P50 was defined as the most positive peak between 40 and 70 ms after stimulus onset, N100 as the most negative peak between 70 and 150 ms, P200 as the most positive peak between 170 and 230 ms, P300 as the most positive peak between 250 and 500 ms, and LPP as the mean amplitude between 500 and 1000 ms. We restricted ERP component analyses to channels Fz, Cz, and Pz because previous studies focused on these midline electrodes as demonstrated in Table 1. For cluster-based ERP analyses, we employed all 11 channels using the FieldTrip toolbox [29].

### Time–frequency analysis

Time–frequency analyses were performed using the FieldTrip toolbox. Epochs were re-extracted from −500 to 1500 ms surrounding stimulus onset. EEG data were converted into the time-frequency domain data using the Morlet wavelet transform (2–40 Hz in steps of 1 Hz). Mean time–frequency representations of each participant and condition were obtained by averaging the time–frequency representations across trials per condition. The ERSP was calculated using the baseline correction approaches of relative change, which was defined by $\left[P(t,f)-\bar{R}(\;f)]/\bar{R}(\;f\right)$, where P(t,f) was the power value at a given timepoint *t* and frequency *f*, and $\bar{R}(\;f)$ was the mean power in the baseline time window. To avoid edge artifacts resulting from the wavelet transform, we used the baseline window from −500 to −200 ms before the stimulus. Finally, we performed cluster-based permutation tests on time-frequency data to examine the stimulus and group effects.

## Statistical analyses

Descriptive data are reported as means and standard deviations (SD). Two sample *T*-tests were used to assess group differences in demographic and behavioral performance data, and chi-square (*χ*^*2*^) tests were used for categorical data. For performance data and ERP components (amplitude and latency of P50, N100, P200, and P300) across and within subtypes, we performed two-way mixed ANOVAs to test the effect of stimulus and group, with stimulus type (standard and deviant) as the within-subject factor, and group (ID and NS) as the between-subject factor. For LPP, two-way mixed ANOVAs were only run for mean amplitude. Given the significant age differences observed between the NS group and subtype 4 (*t* = 2.78, *p* < .01), as well as between NS and subtype 5 (*t* = 2.42, *p* < .05), as detailed in Table S1, age was subsequently included as a covariate in the ERP ANOVA analyses for these two subtypes. To further constrain component analyses, we selected the electrode with maximal peak amplitude or amplitude difference between standard and deviant tones for each component (Fz for P50 and P200, Cz for N100, Pz for P300 and LPP). Post-hoc *t*-tests with and without false discovery rate (FDR) correction were applied to examine the between-group amplitude and latency differences for each ERP component. All statistical analyses were performed with IBM SPSS Statistics (version 26.0).

For cluster-based permutation tests of ERPs and time-frequency data, analyses focused on the time interval spanning from 0 to 1000 ms following stimulus onset, thus covering all aforementioned components. A Monte-Carlo permutation test with 1000 iterations was used to determine the significance probability for all comparisons, with *p*-value .025 as the critical alpha level for two-sided testing. Five pairwise comparisons were conducted for ERPs and time–frequency data, including two stimulus type comparisons (deviant vs. standard for the ID and NS groups) and three group comparisons (ID vs. NS for the standard, deviant, and deviant-standard differences [the latter as a proxy for the group*stimulus type interaction]).

All aforementioned analyses were carried out both across the full ID group and for individual ID subtypes.

## Results

### Participant characteristics

As shown in Table 2, the present sample consisted of 201 individuals diagnosed with insomnia disorder (ID group, 148 females, 73.63%, age: 21–69 years) and 70 normal sleeper controls (NS group, 51 females, 72.86%, range: 20–70 years). No group difference was found in terms of sex (*χ*^*2*^* *= 0.02, *p* = .90) and age (*t *= 0.97, *p* = .33). The insomnia severity index was significantly higher for ID than NS (*t *= 25.78, *p* < .001).

**Table 2.** Mean, standard deviations (SD), and group statistics of demographic and oddball performance data in insomnia disorder (ID) and normal sleepers (NS)

	ID (*N *= 201)	NS (*N *= 70)	*t/χ* ^*2*^	*Sig.* (*p*)
Sex (F/M)	148/53	51/19	0.02	.90
Age (years)	49.36 ± 12.61	47.63 ± 13.76	0.97	.33
ISI	15.39 ± 3.77	2.64 ± 2.85	25.78	<.001***
Overall accuracy (%) #	0.98 ± 0.02	0.98 ± 0.02	−0.51	.61
Standard accuracy (%) #	0.99 ± 0.02	0.99 ± 0.02	−0.38	.71
Deviant accuracy (%) #	0.96 ± 0.05	0.96 ± 0.04	−0.63	.53
Overall reaction time (ms) #	441.77 ± 108.41	435.04 ± 116.02	0.41	.69
Standard reaction time (ms) #	428.71 ± 108.87	421.64 ± 117.17	0.42	.66
Deviant reaction time (ms) #	517.91 ± 118.57	512.20 ± 127.12	0.31	.75

# *N*_ID_ = 183, *N*_NS_ = 58;

*** *p* < .001 level.

Subtyping ID patients according to our previous approach [24], we included 31 individuals of subtype 1 (20 females, 64.52%, age: 27–67 years), 83 individuals of subtype 2 (69 females, 83.13%, age: 21–69 years), 28 individuals of subtype 3 (16 females, 57.14%, age: 27–68 years), 29 individuals of subtype 4 (24 females, 82.76%, age: 37–69 years), and 19 individuals of subtype 5 (11 females, 57.89%, age: 33–68 years). 11 ID patients could not be unambiguously assigned to a single subtype and were thus not included in subtype-specific analyses. Detailed demographic data and group statistics between each subtype and NS group can be found in Table S1.

As reported in the Supplementary Materials, reaction time and accuracy rate analyses included a reduced sample of 183 ID patients and 58 NS controls, due to incomplete response markers in 18 ID patients and 12 NS controls. However, ERPs for correct responses only were virtually indistinguishable from ERPs for all trials irrespective of correctness of response, justifying inclusion of all trials for subsequent ERP and time-frequency analyses.

### ERP components

**Figure 1.**
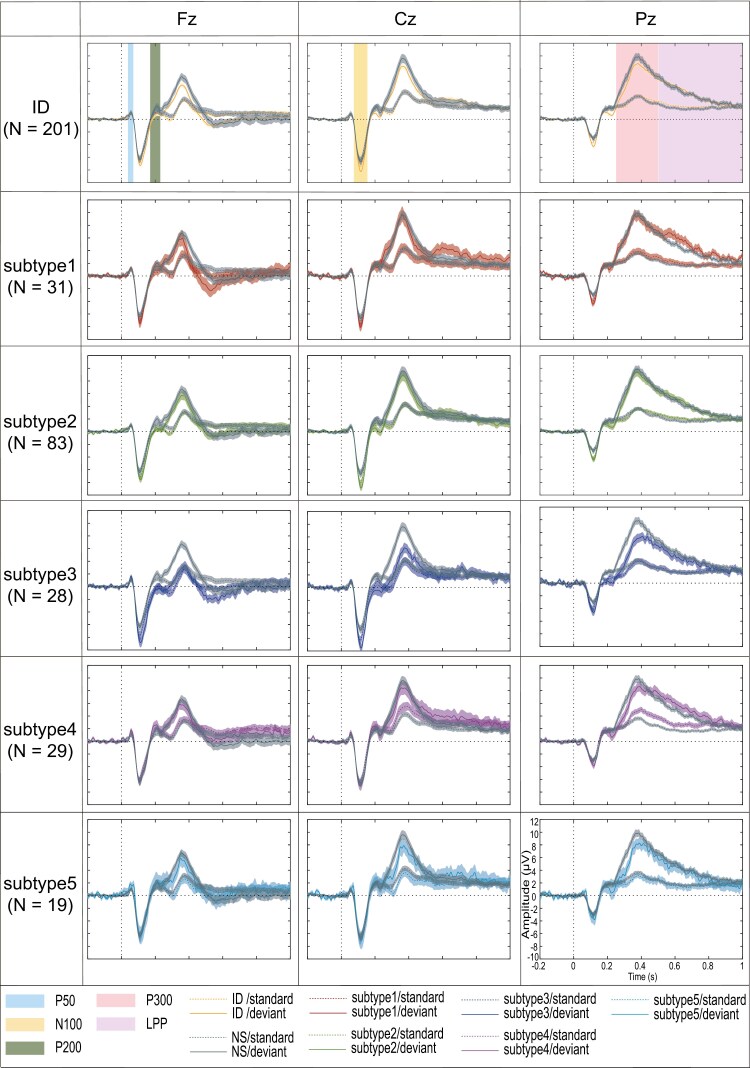
Grand average ERPs at Fz, Cz, and Pz to standard and deviant stimuli in ID group and NS, and five insomnia subtypes.

Figure 1 shows the grand average standard and deviant ERPs at Fz, Cz, and Pz for heterogeneous ID and NS, as well as for different insomnia subtypes, suggesting consistent oddball effects in all cases.

Differences between the heterogeneous ID group and NS for each component’s (P50, N100, P200, P300, LPP) amplitude and latency (except for LPP) were evaluated by nine mixed ANOVAs with factors group and stimulus type. Results are shown in Table S2. For the main effect of stimulus type, we observed significant amplitude and latency effects for all five components (all *p* < .05), with the exception of latency effects for the P200 and P300 components. These results confirmed the overall oddball effect evoked by the task. Regarding the main effect of group, we observed a significant effect on N100 amplitude [*F* (1, 269) = 7.99, *p* = .005, *η*^*2*^*p* = .03]. In addition, we also observed a significant interaction between group and stimulus type on P300 amplitude [*F* (1, 269) = 4.72, *p* = .03, *η*^*2*^*p* = .02]. Post-hoc comparisons (see Table 3) revealed that the ID group exhibited stronger N100 amplitudes both in standard and deviant conditions. However, these group differences for N100 amplitude were no longer significant after FDR correction for multiple comparisons.

**Table 3.** Amplitude and latency means, standard deviations (SD), and group comparison statistics of P50, N100, P200, P300, and LPP at the site of maximal amplitude difference between standard (stand) and deviant (dev) tones in insomnia disorder (ID) and normal sleepers (NS) groups

	Amplitude							Latency						
	ID		NS					ID		NS				
	Mean	SD	Mean	SD	t	*p*	*P*_*fdr*_	Mean	SD	Mean	SD	*t*	*p*	*p*_*fdr*_
P50 (Fz), stand	1.49	1.28	1.30	1.42	1.02	.31	.62	57.09	7.7	57.03	7.76	0.06	.95	.95
P50 (Fz), dev	2.11	1.75	1.98	2.45	0.46	.64	.95	54.95	9.13	54.80	9.76	0.11	.91	.95
N100 (Cz), stand	**−8.34**	**3.66**	**−7.02**	**3.40**	**−2.64**	**.009** **	.08	112.02	11.44	112.17	13.53	−0.09	.93	0.95
N100 (Cz), dev	**−9.52**	**3.97**	**−7.96**	**3.90**	**−2.84**	**.005** **	.08	115.48	13.65	114.97	14.92	0.26	.79	0.95
P200 (Fz), stand	1.64	2.57	2.00	2.57	−1.03	.30	.62	205.33	19.73	206.63	17.94	−0.48	.63	0.95
P200 (Fz), dev	2.64	3.85	3.22	3.96	−1.07	.29	.62	205.77	17.79	205.43	17.12	0.14	.89	0.95
P300 (Pz), stand	4.93	2.51	4.82	2.46	0.32	.75	.95	395.94	64.59	380.00	66.24	1.77	.08	0.36
P300 (Pz), dev	11.07	5.08	12.13	4.76	−−1.53	.13	.47	393.71	52.73	381.09	46.82	1.77	.08	0.36
LPP (Pz), stand	2.08	1.82	1.80	1.70	1.11	.27	.62	—	—	—	—	—	—	—
LPP (Pz), dev	3.71	3.03	3.66	2.39	0.12	.91	.95	—	—	—	—	—	—	—

** *p* < .01 level.

Given these weak and ultimately nonsignificant group difference even in a large sample, we reasoned that clearer deviations might exist when comparing individual ID subtypes to the NS group. Complete ANOVAs results for individual subtypes can be found in Table S3-S7. Regarding the main effect of group (ID subtypes vs NS), significant effects were observed on N100 amplitude and P300 latency in both subtypes 2 and 3, as well as on P200 and P300 amplitudes in subtype 3 (all *p* < .01). Moreover, we also observed significant interactions between stimulus type and group on P300 amplitude in subtype 3 and 4. Post-hoc comparisons for amplitude and latency (see Table 4) revealed that subtypes 2 and 3 displayed stronger N100 amplitudes in comparison to NS controls in both standard and deviant conditions. Subtype 3 additionally exhibited lower P200 and P300 amplitudes, along with a longer P300 latency in the deviant condition. Subtype 4 showed a stronger LPP amplitude for standard tones and a longer P300 latency for deviant tones. However, after FDR correction for multiple comparisons, group differences for P200 amplitude in subtype 3, and LPP amplitude and P300 latency in subtype 4 no longer retained statistical significance.

**Table 4.** Mean (M), standard deviations (SD), and group statistics of P50, N100, P200, P300, and LPP amplitude at site of maximal amplitude difference between standard (stand) and deviant (dev) tones in individual subtypes and normal sleepers (NS) group

	M±SD					Between-group statistics [*t* (*p/p*_*FDR*_)]				
	Subtype 1	Subtype 2	Subtype 3	Subtype 4	Subtype 5	Subtype 1 vs. NS	Subtype 2 vs. NS	Subtype 3 vs. NS	Subtype 4 vs. NS	Subtype 5 vs. NS
**Amplitude**										
P50(Fz), stand	1.63±1.23	1.33±1.18	1.70±1.50	1.85±1.20	1.55±1.11	1.11(0.27/0.94)	0.17(0.86/0.98)	1.26(0.21/0.47)	1.82(0.07/0.29)	0.71(0.48/0.89)
P50(Fz), dev	2.07±1.39	1.94±1.79	2.42±1.74	2.49±1.38	2.69±2.09	0.20(0.84/0.94)	−0.12(0.90/0.98)	0.86(0.39/0.60)	1.05(0.30/0.42)	1.15(0.25/0.89)
N100(Cz), stand	−8.37±3.55	−8.90±3.98	−9.11±3.46	−7.28±2.64	−7.43±3.90	−1.82(0.07/0.81)	**−** **3.11(0.002** ^**^ **/0.02** ^*^)	**−** **2.73(0.007** ^**^ **/0.045** ^*^)	−0.37(0.72/0.72)	−0.45(0.65/0.89)
N100(Cz), dev	−9.31±3.48	−10.04±4.10	−10.56±3.38	−8.25±3.22	−8.74±5.59	−1.66(0.10/0.81)	**−** **3.20(0.002** ^**^ **/0.02** ^*^)	**−** **3.09(0.003** ^**^ **/0.045** ^*^)	−0.36(0.72/0.72)	−0.70(0.49/0.89)
P200(Fz), stand	1.70±2.73	1.23±2.43	0.94±2.69	2.61±2.20	2.64±2.82	−0.54(0.59/0.94)	−1.91(0.06/0.36)	−1.83(0.07/0.18)	1.12(0.27/0.41)	0.94(0.35/0.89)
P200(Fz), dev	3.38±4.10	2.23±3.55	1.42±3.35	3.92±3.72	3.88±5.24	0.19(0.85/0.94)	−1.63(0.11/0.39)	**−** **2.12(0.04** ^*^ **/**0.14)	0.82(0.41/0.53)	0.60(0.55/0.89)
P300(Pz), stand	5.23±3.04	4.83±2.45	4.34±2.62	5.82±2.16	5.02±1.80	0.72(0.47/0.94)	0.03(0.98/0.98)	−0.85(0.40/0.60)	1.91(0.06/0.29)	0.34(0.74/0.89)
P300(Pz), dev	11.83±4.00	11.43±6.05	9.61±3.80	10.85±4.20	11.21±4.60	−0.30(0.76/0.94)	−0.79(0.43/0.79)	**−** **2.50(0.01** ^**^ **/0.045** ^*^)	−1.26(0.21/0.40)	−0.75(0.45/0.89)
LPP(Pz), stand	1.89±1.91	2.03±1.86	1.92±1.62	2.67±1.71	1.73±2.14	0.24(0.81/0.94)	0.78(0.44/0.79)	0.32(0.75/0.87)	**2.29(0.02** ^*^/0.18)	−0.15(0.88/0.91)
LPP(Pz), dev	4.38±2.71	3.65±3.26	3.19±2.67	4.39±2.88	3.09±3.45	1.35(0.18/0.81)	−0.03(0.98/0.98)	−0.84(0.40/0.60)	1.30(0.20/0.40)	−0.83(0.41/0.89)
**Latency**										
P50(Fz), stand	57.81±7.85	56.39±7.87	56.86±7.25	58.21±6.47	57.26±8.65	0.46(0.64/0.94)	−0.51(0.61/0.87)	−0.10(0.92/0.92)	0.72(0.47/0.54)	0.11(0.91/0.91)
P50(Fz), dev	52.90±9.89	54.31±9.14	55.29±8.44	57.93±7.38	57.47±9.82	−0.90(0.37/0.94)	−0.32(0.75/0.96)	0.23(0.82/0.87)	1.55(0.12/0.36)	1.06(0.29/0.89)
N100(Cz), stand	112.39±12.06	114.31±10.46	113.00±9.96	107.03±11.45	110.11±11.26	0.08(0.94/0.94)	1.10(0.27/0.69)	0.29(0.77/0.87)	−1.80(0.08/0.29)	−0.61(0.54/0.89)
N100(Cz), dev	115.35±10.74	116.96±12.46	117.43±11.69	112.69±14.14	115.58±21.70	0.13(0.90/0.94)	0.90(0.37/0.79)	0.78(0.44/0.61)	−0.70(0.48/0.54)	0.14(0.89/0.91)
P200(Fz), stand	209.68±19.59	205.01±20.38	205.43±20.92	202.21±16.72	208.21±19.48	0.77(0.45/0.94)	−0.52(0.61/0.87)	−0.29(0.78/0.87)	−1.14(0.26/0.41)	0.34(0.74/0.89)
P200(Fz), dev	210.32±16.55	204.00±19.14	209.71±17.12	200.00±16.63	207.58±16.32	1.34(0.18/0.81)	−0.48(0.63/0.87)	1.12(0.27/0.54)	−1.45(0.15/0.39)	0.49(0.63/0.89)
P300(Pz), stand	374.97±78.62	398.80±63.13	405.71±48.92	396.55±46.45	396.63±91.70	−0.33(0.74/0.94)	1.79(0.08/0.36)	1.86(0.07/0.18)	1.23(0.22/0.40)	0.89(0.38/0.89)
P300(Pz), dev	379.10±51.51	392.92±48.41	408.71±54.86	411.11±54.23	386.74±60.18	−0.19(0.85/0.94)	1.53(0.13/0.39)	**2.51(0.01** ^**^ **/0.045** ^*^)	**2.80(0.006** ^**^/0.11)	0.44(0.66/0.89)

FDR, family discovery rate. The FDR correction for multiple comparisons was performed within individual subtypes; ^**^p < 0.01 level; ^*^p < 0.05 level.

In sum, the nonsignificantly larger N100 relative to NS in the heterogeneous ID group is driven by ID subtypes 2 and 3, and is diluted by including subtypes 1, 4, and 5 in the sample. Other subtype-specific deviations revealed were strongly diluted in the heterogeneous ID sample, e.g., the lower P300 amplitude and longer P300 latency for deviants in subtype 3.

### ERP clusters

**Figure 2.**
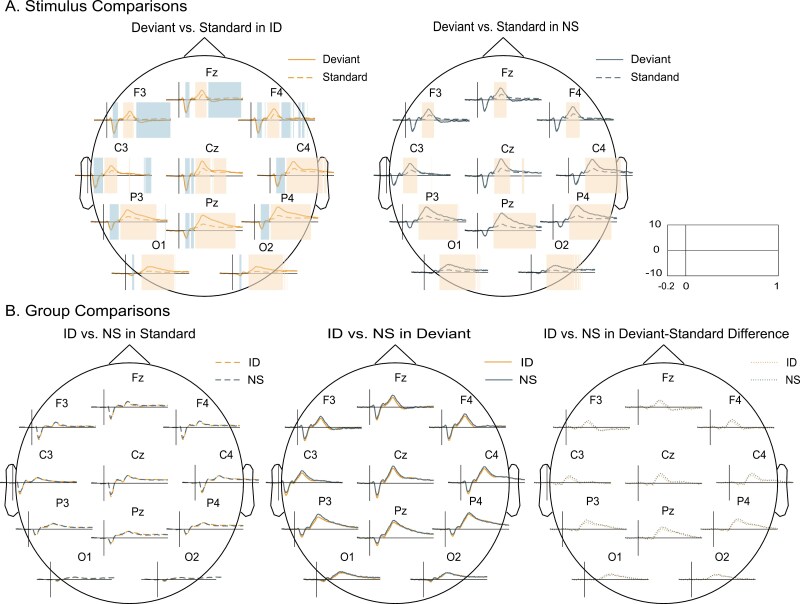
Cluster-based permutation tests to compare the ERPs both in stimulus type and group conditions. Shaded colors indicate time windows with significantly higher (orange) or lower (blue) voltage. (A) In deviants contrasted with standards in ID group (left) and NS (right). Two negative clusters and one positive cluster were observed in ID group (negative cluster 1: *p* = .003, from 100 to 244 ms, 11 channels; positive cluster 1: *p *= .001, from 252-884 ms, 11 channels; negative cluster 2: *p* = .003, from 468 to 1000 ms, 5 channels). One positive cluster was observed in NS (*p* = .001, from 240 to 912 ms, 11 channels). (B) In ID contrasted with NS in standard condition (left), deviant condition (middle), and deviant-standard difference condition (right).

To consider all timepoints across multiple channels rather than predefined ERP components, we next conducted cluster-based permutation tests to compare ERPs between stimulus type and group. As shown in Figure 2, we observed a significant effect of stimulus type, with clusters spanning all channels for both the ID and NS groups. However, contrasting groups, separately for deviants and standards, revealed no significant group effects. Similarly, contrasting deviant-standard difference waves between groups (as a proxy for the group*stimulus type interaction) yielded no significant clusters. Overall, these effects are consistent with the component-based results, showing clear oddball effects but no or negligible group effects.

**Figure 3.**
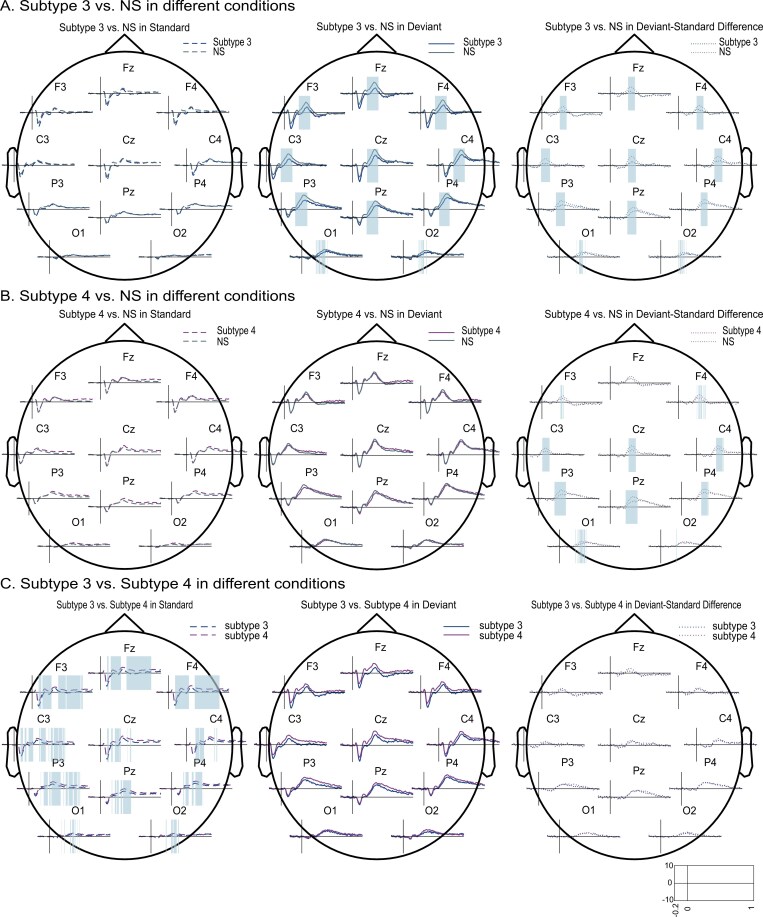
Cluster-based permutation tests to compare the ERPs of subtype 3, subtype 4, and NS in different stimulus-type conditions. Shaded colors indicate time windows with significantly lower (blue) voltage. (A) In subtype 3 contrasted with NS in standard (left), deviant (middle), and deviant-standard difference (right) conditions. One negative cluster was observed in deviant condition (*p* = .006, from 232 to 432 ms, 11 channels). One negative cluster was observed in the deviant-standard difference condition (*p* = .009, from 284 to 432 ms, 11 channels). (B) In subtype 4 contrasted with NS in standard (left), deviant (middle) and deviant-standard difference (right) conditions. One negative cluster was observed in deviant-standard difference condition (*p* = .001, from 256 to 452 ms, 11 channels). (C) In subtype 3 contrasted with subtype 4 in standard (left), deviant (middle) and deviant-standard difference (right) conditions. Two negative clusters were observed in standard condition (negative cluster 1: *p* = .02, from 108 to 360 ms, 11 channels; negative cluster 2: *p* = .01, from 372 to 844 ms, 11 channels).

We next turned to subtype analyses, comparing ERPs of each subtype to those of NS controls. As we expected based on Figure 1, each subtype exhibited significant oddball effects, but only subtypes 3 and 4 showed significant group effects. Specifically, as shown in Figure 3A, ERP amplitude in subtype 3 was significantly lower than that of the NS controls in a window comprising the P300 components for deviants (*p* = .006, from 232 to 432 ms, 11 channels), and for deviant-standard difference waves (*p* = .009, from 284 to 432 ms, 11 channels). Note that these effects echo the P300 observations from the component-based approach. Similarly, as depicted in Figure 3B, subtype 4 demonstrated significantly lower amplitudes compared to NS group for deviant-standard difference waves in the P300 range (*p* = .001, from 256-452 ms, 11 channels). Based on these observations, we also explored direct contrasts between subtypes 3 and 4 (Figure 3C). This revealed a stronger N100 response and attenuated P200/P300 responses for standards in subtype 3 (negative cluster 1: *p* = .02, from 108 to 360 ms, 11 channels; negative cluster 2: *p* = .01, from 372 to 844 ms, 11 channels).

We also conducted cluster-based permutations in the time-frequency domain, where comparisons both across and within subtypes demonstrated significant effects of stimulus type but nonsignificant group effects for standard, deviant, and deviant-standard difference conditions. Detailed results can be found in Supplementary Materials and Figure S3.

In sum, whereas the heterogeneous ID group shows no unequivocal evidence for altered neural responses, subtype analyses suggest diminished P300 oddball effects for subtypes 3 and 4, with these subtypes themselves showing subtle differences in response to standard stimuli.

## Discussion

In the current study, we examined oddball EEG brain activity across a large sample of ID patients, including five previously identified ID subtypes, and NS controls. ERP component analyses with FDR corrections indicated that both subtype 2 and 3 exhibited more negative N100 amplitudes for standards and deviants, while subtype 3 additionally showed lower P300 amplitude and longer P300 latency for deviants. Additionally, cluster-based permutation tests on ERPs further revealed attenuated P300 oddball effects for subtypes 3 and 4. Importantly, when data are pooled across ID subtypes (thus no longer differentiating between ID subtypes), previously observed effects become diluted rather than stronger as would be expected from an increased sample size. These findings support the hypothesis that ID is a heterogeneous disorder wherein attentional brain responses differ depending on insomnia subtype with respect to their neural correlates.

### ERP deviations across heterogeneous ID

We compared the heterogeneous ID and NS groups using both conventional analyses of ERP components and cluster-based approaches across all time-channel (or time-frequency-channel) datapoints. While the component-based approach without correction for multiple comparisons suggested stronger N100 responses in heterogeneous ID relative to NS group, in line with a previous study [17], these deviations did not survive FDR correction for multiple comparisons. Many previous studies (shown in Table 1) did not address the multiple comparisons problem, which may have resulted in false positive reports. Moreover, even among the studies that did account for multiple comparisons, the outcomes are inconsistent. For example, Bastien et al. (2008) observed a statistically significant group difference in N100 amplitude and P200 latency [17], while Turcotte et al. (2011) reported nonsignificant group differences after performing mixed ANOVAs with Bonferroni corrections [18].

Although classical ERP components are well-established and facilitate comparisons to existing literature, ERP component analysis also requires specifying time windows of interest a priori. Cluster-based approaches mitigate some of these challenges, allowing for the identification of ERP effects without predefined components and controlling for multiple comparisons [30]. In our study, cluster-based ERP analyses revealed significant oddball effects in both the ID and NS groups, consistent with the observations from ERP components. However, like the component-based finding with FDR corrections, we did not observe any deviations when comparing the heterogeneous ID group with NS controls. Similarly, whereas cluster-based time-frequency analyses indicated clear oddball effects for both groups, no significant group differences were found.

All told, even with a substantial sample our results suggest very limited evidence for systematically altered oddball processing occurring uniformly across all people with ID in the same way, irrespective of subtype.

### Subtype-specific ERP responses

Next to comparing heterogeneous ID with NS, we evaluated deviations separately for all five ID subtypes. ERP component analyses without corrections for multiple comparisons revealed stronger N100 amplitudes for standards and deviants in subtypes 2 and 3, lower P200 and P300 amplitudes for deviants in subtype 3, longer P300 latencies in subtypes 3 and 4, and higher LPP amplitude for standards in subtype 4. However, after FDR corrections for multiple comparisons, group differences for P200 amplitude in subtype 3, and higher LPP amplitude and longer P300 latency in subtype 4 no longer reached significance. Moreover, cluster-based ERP analyses indicated significant P300 amplitude attenuations in subtypes 3 (for the deviant and deviant-standard difference conditions) and 4 (for the deviant-standard difference condition). Hence, the diminished P300 amplitude in subtype 3 was seen across both component-based and cluster-based ERP analyses, providing additional support for this finding.

Numerous studies have identified hyperarousal as a central construct in the pathophysiology of ID [5–7, 9, 10, 31]. The neurocognitive model of insomnia [7] posits that conditioned arousal of the central nervous system results in maladaptively enhanced sensory and information processing. Furthermore, the hyperarousal model delineates increased sensitivity to sensory stimuli as a state marker of hyperarousal in insomnia [5]. Considering the ERP P50 as a detector of early sensory gating [32, 33], and the associations of N100 and P200 components with arousal levels [34] and selective attention [35, 36], the possible ERP manifestations of hyperarousal may involve abnormalities in these ERP components. Contrary to these ideas, we did not observe significant P50 amplitude and latency effects, and only nonsignificant P200 amplitude effects in subtype 3 after FDR correction. However, concerning the N100, we did observe abnormal amplitude effects in both subtypes 2 and 3. The stronger N100 amplitudes relative to NS controls in subtypes 2 and 3 suggest that task-specific hyperarousal is reflected by early attentional processes rather than early sensory gating.

In addition to these components reflecting early perceptual and attentional processing, the P300 and LPP are the ERP components that relate to sustained attention and emotional processing [37, 38]. The P300 is known to reflect the allocation of capacity-limited attentional resources toward task-relevant stimuli [38–40]. Additionally, it is proposed as an ERP manifestation of the phasic locus coeruleus-norepinephrine (LC-NE) response bursts [41], a mechanism that facilitates the exploitation of the current environment and optimizes task performance [42, 43]. Similarly, the LPP appears to reflect the sustained increase in attention toward emotionally arousing stimuli and the processing thereof [37, 44], and it is hypothesized to be generated via the LC-NE system in response to emotional stimuli [37]. Given the well-understood associations of the LC-NE system with arousal and task-related decision processes [43], and the frequent reports of hyperarousal [5, 9] and cognitive deficits [4] in insomnia disorder, it might be expected that people with insomnia may present alterations in P300 and LPP linked to impaired attentional and emotional processing.

In our study, we observed lower P300 amplitude and longer P300 latency for deviants in subtype 3, alongside a smaller difference between deviant and standard P300 amplitudes in subtypes 3 and 4. Subtype 3 is primarily characterized by reward insensitive and reduced experience of pleasure, while subtype 4 is characterized by high reactivity to environment stimuli and life events [24]. The attenuated P300 amplitude and longer P300 latency for deviants in subtype 3 suggests their reduced sensitivity to reward, or surprise, and could be associated with a reduced discrimination between common and uncommon stimuli. The diminished P300 amplitude for deviant-standard differences in subtype 4, however, may indicate an improperly balanced allocation of attentional resources towards different stimuli. This imbalance could result in the perception of standard tones as more salient and less distinguishable from deviant tones, in agreement with this subtype’s highly reactive nature. Regarding the LPP component, while we initially observed that subtype 4 exhibits stronger LPP amplitude for standards, this effect failed to maintain significance following FDR correction and cluster-based permutations on ERPs. Consequently, we remain uncertain as to whether there exists a reduction of LPP amplitude in subtype 4.

Taken together, these findings determine insomnia subtype-specific ERP deviations occur, and show how these deviations are diluted in heterogeneous samples. The more pronounced N100 amplitudes observed in subtypes 2 and 3 could potentially reflect task-specific hyperarousal within these subtypes. The diminished P300 amplitudes observed in subtypes 3 and 4 may reflect the impaired allocation of attentional resources towards different stimuli within these subtypes.

## Limitations

There are several limitations to the current study. First, the auditory oddball paradigm is a relatively simple task, and patients with ID may be more sensitive to detect abnormal attentional brain activity under specific conditions, e.g. when exposed to emotional stimuli. Further studies could consider more sensitive tasks in different cognitive and emotional domains. Second, in the current study, we investigated the trait-like ERP deviations between ID and NS controls during pre-sleep wakefulness. However, ERP characteristics could vary across days in relation to sleep quality as reported in previous studies [14, 15]. Future studies could explore this state-like aspect of ERP responses in particular insomnia subtypes. Finally, while we tested the a priori hypothesis that oddball ERP alterations are specific to certain ID subtypes, we did not have hypotheses regarding how each ERP component might be affected in each subtype. Therefore, subtype-specific analyses may be best characterized as “secondary analysis of a confirmatory data analysis using exploratory data analysis,” and interpretation should be done with caution [45].

## Conclusion

Our findings reveal that ERPs are altered in different ways for different ID subtypes and that pooling across subtypes dilutes the effects present in specific subtypes. In a heterogeneous sample of ID, even if it is large, group differences with NS controls are weak and ultimately nonsignificant. Enhanced N100 amplitudes are only seen in subtypes 2 and 3, but not in subtypes 1, 4, and 5. A lower P300 amplitude and longer P300 latency for deviants occurred in subtype 3 only. A smaller difference between deviant and standard P300 amplitudes were seen only in subtypes 3 and 4. Candidate neural markers of task-specific hyperarousal may be sought in the N100 component related to early attentional processing (in subtypes 2 and 3). The impaired allocation of attentional resources toward different stimuli may be sought in P300 component (in subtypes 3 and 4). Our study emphasizes the relevance of subtyping insomnia when investigating different brain correlates of insomnia vulnerability, which can be done online (https://staging.insomniatype.org/).

## Supplementary material

Supplementary material is available at *SLEEP* online.

zsaf056_suppl_Supplementary_Figures_S1-S3_Tables_S1-S7

## Data Availability

The dataset supporting the conclusions of this article is available from the corresponding author, upon reasonable request. Open Science online subtyping available on https://staging.insomniatype.org/
