# Supplementary material for: One size fits null: attentional brain responses differ depending on insomnia subtype
Source: Sleep. 2025 May 22;48(7):zsaf056. doi: 10.1093/sleep/zsaf056 (PMC12246374; doi:10.1093/sleep/zsaf056)
Supplement: zsaf056_suppl_Supplementary_Figures_S1-S3_Tables_S1-S7 [file zsaf056_suppl_supplementary_figures_s1-s3_tables_s1-s7.pdf]

Supplementary Materials

One size fits null: attentional brain responses differ depending on insomnia subtype

Wenrui Zhao[1,2], Eus J.W. Van Someren[2,3,4], Glenn van der Lande[5,6], Sjors van de Ven[2,7], Frank J.van Schalkwijk[2,8], Tessa F Blanken[9], Jennifer R. Ramautar[2,10,11,12*], Roy Cox[2*]

[1] Sleep Medicine Center, Chongqing Traditional Chinese Medicine Hospital, Chongqing, 400021, China

[2] Department of Sleep and Cognition, Netherlands Institute for Neuroscience, An Institute of the Royal Netherlands Academy of Arts and Sciences, Amsterdam, The Netherlands

[3] Department of Psychiatry, Amsterdam Public Health Research Institute and Amsterdam Neuroscience Research Institute, Amsterdam UMC, Vrije Universiteit, The Netherlands

[4] Department of Integrative Neurophysiology, Center for Neurogenomics and Cognitive Research (CNCR), Amsterdam Neuroscience, Vrije Universiteit Amsterdam, The Netherlands

[5] Coma Science Group, GIGA-Consciousness, University of Liège, Liège, Belgium

[6] Centre du Cerveau2, University Hospital of Liège, Liège, Belgium

[7] Department of Biological Psychology, Vrije Universiteit Amsterdam, The Netherlands

[8] Hertie-Institute for Clinical Brain Research, Center for Neurology, University Medical Center Tübingen, Otfried-Müller Str. 27, 72076 Tübingen, Germany

[9] Department of Psychology, University of Amsterdam, The Netherlands

[10] N=You Neurodevelopmental Precision Center, Amsterdam Neuroscience, Amsterdam Reproduction and Development, Amsterdam UMC, Amsterdam, The Netherlands

[11] Child and Adolescent Psychiatry and Psychosocial Care, Emma Children's Hospital, Amsterdam UMC, Vrije Universiteit Amsterdam, Amsterdam, The Netherlands

[12] Emma Center for Personalized Medicine, Amsterdam UMC, Amsterdam, The Netherlands

[*] Jennifer R. Ramautar and Roy Cox share senior authorship

Corresponding Author

Eus J.W. Van Someren, Ph.D.

Department of Integrative Neurophysiology,

Center for Neurogenomics and Cognitive Research (CNCR),

Neuroscience Campus Amsterdam,

VU University Amsterdam,

De Boelelaan 1085

1081 HV Amsterdam

The Netherlands

Email: e.j.w.someren@vu.nl

Supplementary Results

Results

Oddball performance (183 vs. 58)

Due to incomplete response markers, performance data analyses included a reduced sample of 183 ID patients and 58 NS controls. Accuracy was nearly at ceiling and did not differ between groups in pairwise contrasts (Table 2). In an ANOVA considering both group and stimulus type, we observed a significant main effect of stimulus type in accuracy rate [F (1, 239) = 70.76, $p < 0.001$, $\eta^2p = 0.23$], with deviants having a slightly reduced accuracy. Similarly, reaction times did not differ between groups in pairwise contrasts, while the ANOVA yielded a significant main effect of stimulus type [F (1, 239) = 347.06, $p < 0.001$, $\eta^2p = 0.59$], with deviants showing a ~90 ms longer reaction time. Performance results for different subtypes were very similar (Table S1).

ERP control analyses for subsample (183 vs. 58)

To assess whether meaningful ERP and time-frequency analyses could be performed when recordings lack response markers (and consequently, trial accuracy information), we first conducted control analyses on the subsample with trial accuracy information. Specifically, we compared grand average ERPs of trials with correct responses to ERPs of all trials, separately for standard and deviant stimuli, and for channels Fz, Cz, and Pz. Owing to the aforementioned high oddball performance, ERPs for correct vs. all trials were virtually indistinguishable for both stimulus types (Figure S2), justifying inclusion of the entire sample for subsequent ERP and time-frequency analyses regardless of trial accuracy.

Time-frequency analyses

we conducted cluster-based analyses in the time-frequency domain. As illustrated in Figure S3, consistent with the finding from ERP analyses, time-frequency analyses

revealed significant effects of stimulus type in both ID (positive cluster 1: p = 0.001, higher 2-15 Hz power in the range of 12 ms to 1000 ms across 11 channels; positive cluster 2: p = 0.002, higher 13-40 Hz power in the range of 236 ms to 1000 ms, across 11 channels; negative cluster 1: p = 0.001, lower 5-27 Hz power in the range of 172 ms to 1000 ms across 11 channels) and NS groups (positive cluster 1: p = 0.001, higher 2-10 Hz power in the range of 12 ms to 1000 ms across 11 channels; negative cluster 1: p = 0.002, higher 6-29 Hz power in the range of 236 ms to 1000 ms across 11 channels) (Figure S3A), while no significant group effect was observed for standards, deviants, and the deviant-standard differences (Figure S3B). Comparisons between each ID subtype and the NS group demonstrated analogous outcomes: significant effects of stimulus type, but no significant group effects for standard, deviant, and deviant-standard difference conditions.

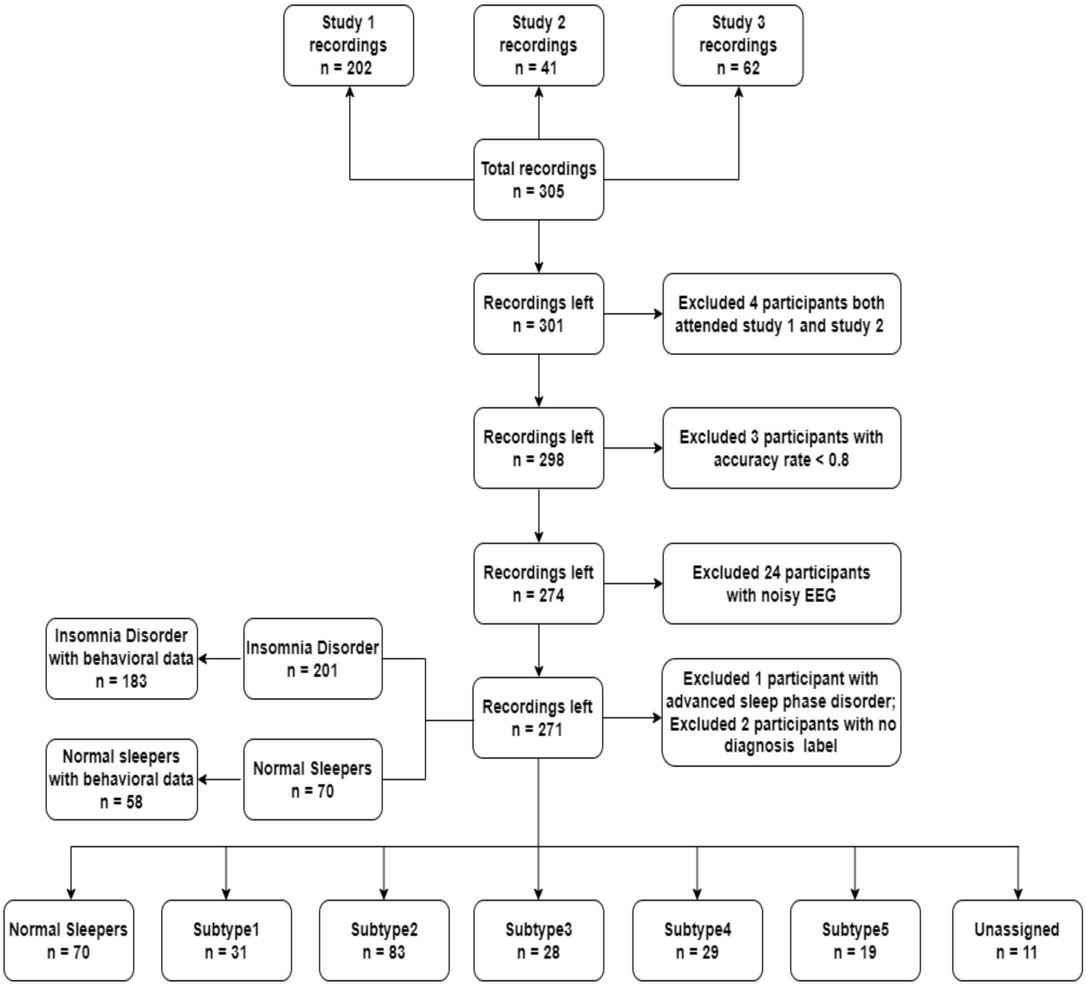

Figure S1. The flowchart of data selection

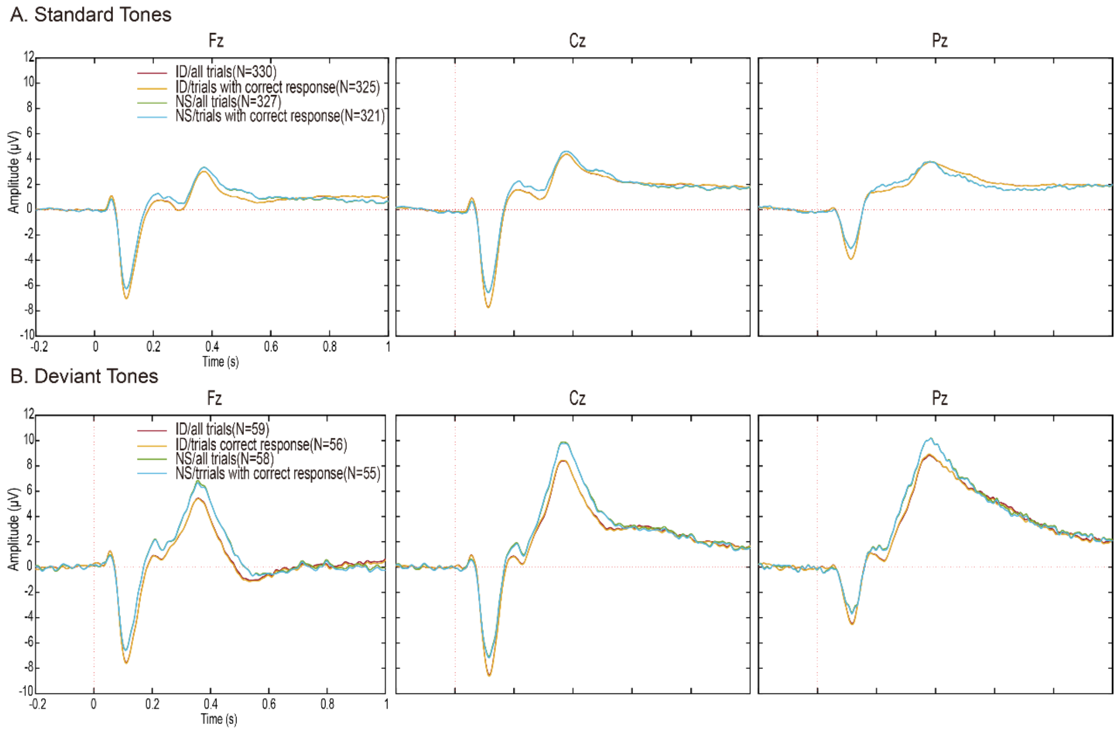

Figure S2. Control analyses of grand average ERPs for all trials and trials with correct response to standard and deviant stimuli at Fz, Cz, and Pz

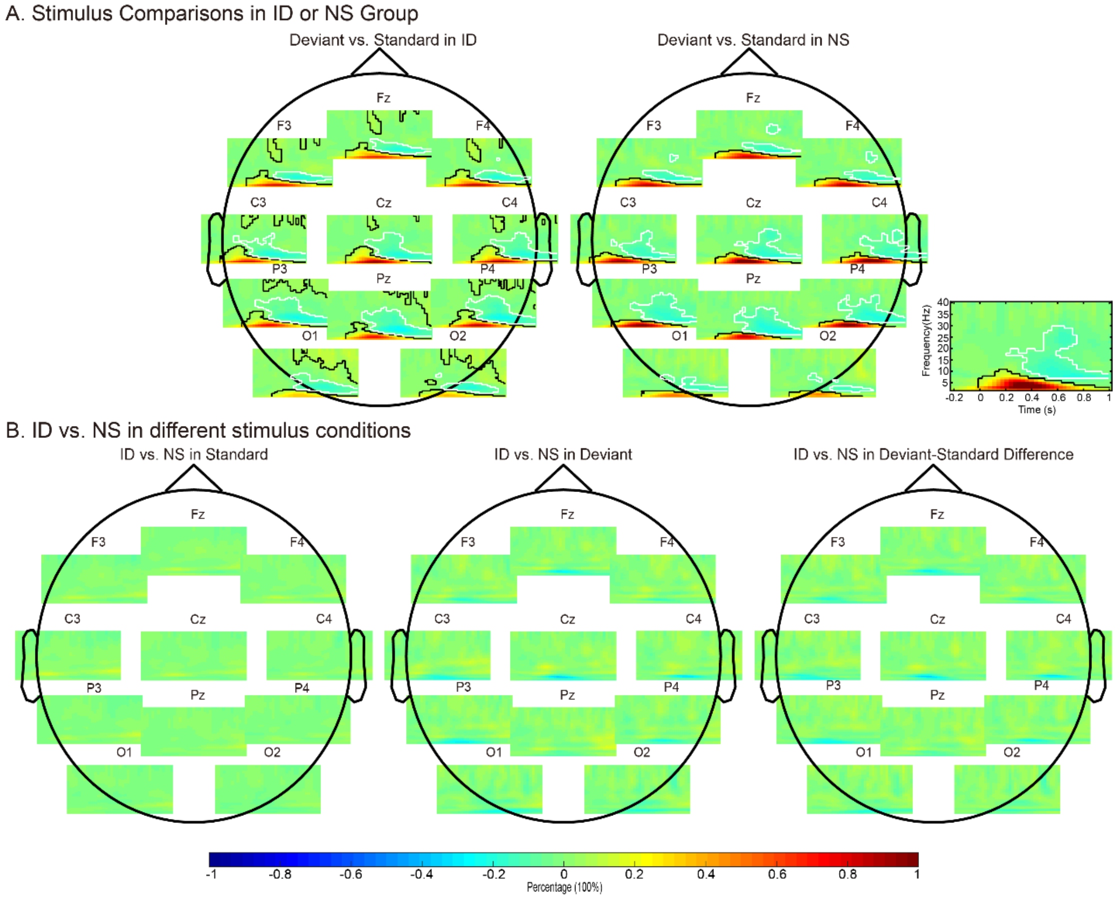

Figure S3. Cluster-based permutation tests to compare the time frequency

representations in different group conditions and different stimulus conditions. Positive clusters are marked in black lines, and negative clusters are marked in white lines. (A) In deviants contrasted with standards in ID (left) and NS group (right). Two positive clusters and one negative cluster were observed in ID group (positive cluster 1: p = 0.001, from 12-1000 ms, from 2-15 Hz, 11 channels; negative cluster 1: p = 0.002, from 12-1000 ms, from 2-15 Hz, 11 channels; positive cluster 2: p = 0.001, from 236-1000 ms, from 13-40 Hz, 11 channels). One positive cluster and one negative cluster were observed in NS group (positive cluster 1: p = 0.001, from 12-1000 ms, from 2-10 Hz, 11 channels; negative cluster 1: p = 0.001, from 236-1000 ms, from 6-29 Hz, 11 channels). (B) In ID contrasted with NS in standard condition (left), deviant condition (middle), and deviant-standard difference condition (right).

Table S1. Mean, standard deviations (SD), and group statistics of demographic and oddball performance data in different insomnia subtypes and normal sleepers (NS)

	NS (N = 70)	Subtype 1 (N = 31)	Subtype 2 (N = 83)	Subtype 3 (N = 28)	Subtype 4 (N = 29)	Subtype 5 (N = 19)	Subtype 1 vs NS	Subtype 2 vs NS	Subtype 3 vs NS	Subtype 4 vs NS	Subtype 5 vs NS
	M ± SD	M ± SD	M ± SD	M ± SD	M ± SD	M ± SD	t/χ^2	t/χ^2	t/χ^2	t/χ^2	t/χ^2
Sex(F/M)	51/19	20/11	69/14	16/12	24/5	11/8	0.72	2.37	2.28	1.10	1.58
Age(years)	47.63 ± 13.76	44.77 ± 11.89	48.42 ± 13.74	48.50 ± 12.70	55.17 ± 7.61	55.84 ± 10.38	-1.00	0.36	0.29	**2.78****	**2.42***
ISI	2.64 ± 2.85	16.52 ± 3.78	15.29 ± 3.64	16.07 ± 3.82	14.54 ± 3.51	14.13 ± 3.59	**20.34*****	**23.60*****	**19.03*****	**17.42*****	**14.70*****
Overall accuracy (%) #	0.98 ± 0.02	0.98 ± 0.04	0.99 ± 0.02	0.98 ± 0.01	0.98 ± 0.01	0.98 ± 0.02	-1.33	0.26	-0.89	-0.17	-0.30
Standard accuracy (%) #	0.99 ± 0.02	0.98 ± 0.03	0.99 ± 0.01	0.99 ± 0.01	0.99 ± 0.01	0.99 ± 0.02	-1.24	0.41	-0.59	0.13	-0.37
Deviant accuracy (%) #	0.96 ± 0.04	0.95 ± 0.07	0.96 ± 0.05	0.95 ± 0.04	0.95 ± 0.05	0.96 ± 0.03	-0.97	-0.25	-1.07	-0.79	0.10
Overall RT (ms) #	435.04 ± 116.02	455.12± 145.97	429.79 ± 96.92	453.43 ± 99.34	438.17 ± 101.99	448.21 ± 126.73	0.69	-0.29	0.71	0.12	0.40
Standard RT (ms) #	421.64 ± 117.17	443.92 ± 147.82	416.53 ± 97.44	440.71 ± 99.54	425.12 ± 105.07	431.43 ± 120.71	0.76	-0.28	0.73	0.13	0.30
Deviant RT (ms) #	512.20 ± 127.12	520.25 ± 145.91	506.77 ± 108.54	527.99 ± 111.50	515.79 ± 100.45	545.16 ± 163.17	0.26	-0.27	0.55	0.13	0.88

Note：# N_{NS}=58，$N_{subtype\ 1}$=28，$N_{subtype\ 2}$=77，$N_{subtype\ 3}$=27，$N_{subtype\ 4}$=28，$N_{subtype\ 5}$= 17; * $P < 0.05$, ** $P < 0.01$, *** $P < 0.001$.

Table S2. Analysis of variance for ERPs amplitude and latency for P50, N100, P200, P300, and LPP at site of maximal peak amplitude or amplitude difference between standard and deviant tones in patients with insomnia disorder (ID) and normal sleeper controls (NS)

	Amplitude			Latency		
	F	*p*	η^2p	*F*	*p*	η^2p
P50 (Fz)						
Group (G)	0.59	0.44	0.002	0.01	0.91	<0.001
Stimulus Type (S)	33.91	**<0.001*****	0.11	9.21	**0.003****	0.03
S × G	0.07	0.79	<0.001	0.003	0.96	<0.001
N100 (Cz)						
Group (G)	7.99	**0.005****	0.03	0.01	0.91	<0.001
Stimulus Type (S)	72.32	**<0.001*****	0.21	14.81	**<0.001*****	0.05
S × G	0.94	0.33	0.003	0.17	0.68	0.001
P200 (Fz)						
Group (G)	1.30	0.26	0.005	0.50	0.82	<0.001
Stimulus Type (S)	32.84	**<0.001*****	0.11	0.07	0.79	<0.001
S × G	0.29	0.59	0.001	0.34	0.56	0.001
P300 (Pz)						
Group (G)	0.99	0.32	0.004	4.45	**0.04***	0.02
Stimulus Type (S)	618.13	**<0.001*****	0.70	0.02	0.90	<0.001
S × G	4.72	**0.03***	0.02	0.14	0.71	0.001
LPP (Pz)						
Group (G)	0.30	0.59	0.001	-	-	-
Stimulus Type (S)	134.27	**<0.001*****	0.33	-	-	-
S × G	0.58	0.45	0.002	-	-	-

Table S3. Analysis of variance for ERPs amplitude and latency for P50, N100, P200, P300, and LPP in subtype 1 and normal sleeper controls (NS)

	Amplitude			Latency		
	F	*p*	$\eta^2 p$	*F*	*p*	$\eta^2 p$
P50 (Fz)						
Group (G)	0.37	0.55	0.004	0.14	0.71	0.001
Stimulus Type (S)	9.34	**0.003****	0.09	8.71	**0.004****	0.08
S × G	0.40	0.53	0.004	1.22	0.27	0.01
N100 (Cz)						
Group (G)	3.19	0.08	0.03	0.01	0.91	<0.001
Stimulus Type (S)	26.42	**<0.001*****	0.21	5.03	**0.03***	0.05
S × G	<0.001	0.99	<0.00	0.004	0.95	<0.001
P200 (Fz)						
Group (G)	0.01	0.92	<0.001	1.50	0.22	0.02
Stimulus Type (S)	20.72	**<0.001****	0.17	0.02	0.89	<0.001
S × G	0.54	0.46	0.005	0.21	0.65	0.002
P300 (Pz)						
Group (G)	0.007	0.94	<0.001	0.11	0.74	<0.001
Stimulus Type (S)	378.07	**<0.001*****	0.79	0.12	0.73	0.001
S × G	0.98	0.32	0.01	0.04	0.84	<0.001
LPP (Pz)						
Group (G)	0.91	0.34	0.009	-	-	-
Stimulus Type (S)	133.71	**<0.001*****	0.58	-	-	-
S × G	2.82	0.10	0.03	-	-	-

Table S4. Analysis of variance for ERPs amplitude and latency for P50, N100, P200, P300, and LPP in subtype 2 and normal sleeper controls (NS)

	Amplitude			Latency		
	F	*p*	η^2p	*F*	*p*	η^2p
P50 (Fz)						
Group (G)	0.00	0.99	<0.001	0.26	0.61	0.002
Stimulus Type (S)	21.35	**<0.001**	0.12	6.24	**0.01****	0.04
S × G	0.08	0.78	0.001	0.008	0.93	<0.001
N100 (Cz)						
Group (G)	10.53	**0.001****	0.07	1.23	0.27	0.008
Stimulus Type (S)	48.98	**<0.001*****	0.245	8.82	**0.003****	0.06
S × G	0.46	0.49	0.003	0.01	0.94	<0.001
P200 (Fz)						
Group (G)	3.60	0.06	0.023	0.36	0.55	0.002
Stimulus Type (S)	23.71	**<0.001*****	0.14	0.43	0.51	0.003
S × G	0.23	0.64	0.001	0.003	0.96	<0.001
P300 (Pz)						
Group (G)	0.34	0.56	0.002	4.09	**0.05***	0.03
Stimulus Type (S)	390.001	**<0.001*****	0.72	0.21	0.65	0.001
S × G	1.02	0.31	0.007	0.44	0.51	0.003
LPP (Pz)						
Group (G)	0.10	0.76	0.001	-	-	-
Stimulus Type (S)	88.87	**<0.001*****	0.37	-	-	-
S × G	0.42	0.52	0.003	-	-	-

Table S5. Analysis of variance for ERPs amplitude and latency for P50, N100, P200, P300, and LPP in subtype 3 and normal sleeper controls (NS)

	Amplitude			Latency		
	F	*p*	η^2p	*F*	*p*	η^2p
P50 (Fz)						
Group (G)	1.23	0.27	0.01	0.01	0.92	<0.001
Stimulus Type (S)	13.51	**<0.001**	0.12	2.50	0.12	0.03
S × G	0.01	0.93	<0.001	0.008	0.79	<0.001
N100 (Cz)						
Group (G)	9.08	**0.003****	0.09	0.37	0.55	0.004
Stimulus Type (S)	35.25	**<0.001*****	0.27	7.98	**0.006****	0.07
S × G	1.61	0.21	0.02	0.41	0.53	0.004
P200 (Fz)						
Group (G)	4.76	**0.03***	0.05	0.22	0.64	0.002
Stimulus Type (S)	7.08	**0.009****	0.07	0.47	0.50	0.005
S × G	1.35	0.25	0.01	1.47	0.23	0.02
P300 (Pz)						
Group (G)	4.28	**0.04***	0.04	7.21	**0.009****	0.07
Stimulus Type (S)	283.59	**<0.001*****	0.75	0.07	0.79	0.001
S × G	7.53	**0.007****	0.07	0.02	0.90	<0.001
LPP (Pz)						
Group (G)	0.16	0.69	0.002	-	-	-
Stimulus Type (S)	59.88	**<0.001*****	0.38	-	-	-
S × G	2.10	0.15	0.02	-	-	-

Table S6. Analysis of variance for ERPs amplitude and latency for P50, N100, P200, P300, and LPP in subtype 4 and normal sleeper controls (NS)

	Amplitude			Latency		
	F	*p*	η^2p	*F*	*p*	η^2p
P50 (Fz)						
Group (G)	0.87	0.35	0.09	1.32	0.25	0.02
Stimulus Type (S)	3.29	0.07	0.03	4.85	**0.03***	0.05
S × G	0.02	0.89	<0.001	2.49	0.12	0.03
N100 (Cz)						
Group (G)	0.23	0.64	0.002	1.59	0.21	0.02
Stimulus Type (S)	0.94	0.34	0.01	0.05	0.82	0.001
S × G	0.28	0.60	0.003	0.34	0.56	0.003
P200 (Fz)						
Group (G)	0.26	0.61	0.003	1.98	0.16	0.02
Stimulus Type (S)	5.35	**0.02***	0.05	2.29	0.13	0.02
S × G	0.26	0.61	0.003	0.37	0.54	0.004
P300 (Pz)						
Group (G)	0.06	0.81	0.001	1.61	0.21	0.02
Stimulus Type (S)	37.03	**<0.001*****	0.28	0.70	0.41	0.007
S × G	5.52	**0.02***	0.05	1.23	0.27	0.01
LPP (Pz)						
Group (G)	2.43	0.12	0.03	-	-	-
Stimulus Type (S)	15.96	**<0.001*****	0.14	-	-	-
S × G	0.06	0.80	0.001	-	-	-

Table S7. Analysis of variance for ERPs amplitude and latency for P50, N100, P200, P300, and LPP in subtype 5 and normal sleeper controls (NS)

	Amplitude			Latency		
	F	*p*	η^2p	*F*	*p*	η^2p
P50 (Fz)						
Group (G)	0.42	0.52	0.01	0.25	0.62	0.003
Stimulus Type (S)	6.22	**0.02***	0.07	2.15	0.15	0.02
S × G	1.78	0.19	0.02	1.57	0.21	0.02
N100 (Cz)						
Group (G)	0.33	0.57	0.004	0.13	0.72	0.002
Stimulus Type (S)	0.77	0.38	0.009	0.32	0.57	0.004
S × G	0.10	0.92	<0.001	0.51	0.48	0.006
P200 (Fz)						
Group (G)	0.11	0.74	0.001	0.35	0.56	0.004
Stimulus Type (S)	6.41	0.01	0.07	1.67	0.20	0.02
S × G	0.22	0.64	0.003	0.04	0.84	<0.001
P300 (Pz)						
Group (G)	0.002	0.96	<0.001	0.03	0.87	<0.001
Stimulus Type (S)	40.78	**<0.001*****	0.32	1.01	0.32	0.01
S × G	0.41	0.52	0.005	0.08	0.77	0.001
LPP (Pz)						
Group (G)	0.75	0.39	0.01	-	-	-
Stimulus Type (S)	13.54	**<0.001*****	0.14	-	-	-
S × G	0.20	0.65	0.002	-	-	-
